# Downconversion Master Slave OCT With a Bidirectional Sweeping Laser

**DOI:** 10.1002/jbio.202400201

**Published:** 2024-08-27

**Authors:** A. Martinez Jimenez, R. Cernat, A. Bradu, R. Riha, E. A. Proano Grijalva, B. O. Meyer, T. Ansbaek, K. Yvind, A. Podoleanu

**Affiliations:** ^1^ Applied Optics Group School of Physical Sciences, University of Kent Canterbury UK; ^2^ DTU Electro, Department of Electrical and Photonics Engineering Technical University of Denmark (DTU) Kgs. Lyngby Denmark; ^3^ OCTLIGHT ApS Kgs. Lyngby Denmark

**Keywords:** bidirectional, OCT, swept‐source

## Abstract

This paper explores the challenges of signal processing when using optical coherence tomography (OCT) imaging instruments driven by asymmetric MHz bidirectional sweeping lasers. A downconversion master–slave (DMS) method is proposed as a viable alternative to the traditional OCT protocol. Unlike conventional swept source OCT, which requires a separate calibration for each sweep, the DMS approach does not require calibration of the acquired channeled spectra; its operation is independent of the tuning direction. We demonstrate the practicality of the DMS method with en‐face OCT images obtained with an OCT instrument equipped with a fast bidirectional swept laser (tuning speed 1.6 MHz) and a slow acquisition card of only 2.5 MS/s sampling rate.

## 1 Introduction

Two optical coherence tomography (OCT) methods are known for processing the spectrum at the OCT interferometer output: spectrometer (Sp) based and swept source (SS) based. Sp‐OCT is limited by the speed of linear cameras used, with demonstrations of up to 312 kHz in the rate of spectrum interrogation [1]. Although there are alternatives to overcome this issue, such as using multiple spectrometers, the complexity and cost of multi‐spectrometer systems render such systems too expensive and difficult to calibrate [2]. SS‐OCT technology has evolved from a few Hz [3] axial scans per second (i.e., A‐Scan rate) to multi‐MHz sweeping rates [4]. Imaging performance and speed improvements in SS‐OCT have been largely depended on the development of the light sources. Moreover, SS‐OCT has shown similar sensitivity to that delivered by Sp‐OCT but larger imaging depth [5, 6, 7, 8], and speed [9]. With improvement in both speed and imaging depth, new applications are achievable, such as real‐time surgical guidance [10], widefield OCT imaging, and delivering volumetric images less affected by the sample movement (such as micro‐saccades in the eye, which are essential for optometry and ophthalmology). Faster imaging reduces phase variations, which is crucial in applications where phase instability may obscure tiny axial displacement [11, 12, 13, 14] or where phase processing is essential such as in OCTA.

Among the many solutions researched for fast tunability, two main categories can be distinguished: akinetic and nonakinetic (involving nonmoving or moving elements, respectively). For the time being, nonakinetic solutions dominate the landscape of SSs. Two main examples in this respect are represented by the micro‐electromechanical systems with vertical‐cavity surface‐emitting lasers (MEMS–VCSEL) [14] and the Fourier Domain mode‐locked (FDML) lasers concepts [15, 16]. In these systems, a mirror is moved within a Fabry–Perot cavity, which produces tuning. Compared to other tunable sources, the MEMS–VCSEL sources exhibit a short cavity. Their single‐mode operation and narrow instantaneous line widths enable long axial range in OCT images.

A SS can sweep from red to blue or blue to red, that is, forward or backward sweep. “Unidirectional sweeping” refers to the use of only one of the sweeps, as opposed to both. The majority of SSs on the market are unidirectional, some with duty ratios less than 50%. Making the tuning bidirectional increases the duty ratio and doubles the tuning rates. However, high‐speed SSs and bidirectional multi‐MHz MEMS–VCSELs provide two fundamental challenges that need to be addressed prior to imaging:

- The ability to use forward and backward sweeping demands precise phase corrections due to the asymmetry in the two tuned spectra [17]. Therefore, signal processing requires different phase corrections for forward and backward sweeping to be applied during each sweep [18].
- Another problem for unidirectional and bidirectional sweeping lasers is that the high sweeping rate demands high‐speed digitizers, and complex software to display the image in real time. Because of the narrow line width of these sources, a long axial range exceeding many centimeters becomes attainable. Therefore, a densely modulated channeled spectrum (CS) may be generated at the interferometer output. This can lead to a radio frequency (RF) spectrum of many GHz of the photo‐detected signal. A high‐speed digitizer required to sample 2 GHz can exceed $10 000. This increases the cost of fast‐sweeping solutions, limiting fast OCT technology to research purposes only.

Yet another concern is the instability in the tuning curve of the SS, which requires frequent calibration. Using *k*‐clocks can address the nonlinearity variation over time, but that is only possible if digitizers with input for *k*‐clocks are used. Hence, new OCT protocols [19, 20, 21] are needed to address or alleviate the problems listed above.

In this paper, we present a solution to address both challenges raised by the high sweep rates and those raised by bidirectional sweeping. Using a second interferometer for any depth of interest, driven by the same SS, as for the OCT interferometer, signal with similar chirpt to that in the OCT interferometer is created. By multiplying the photodetected signals delivered by the two interferometers, depth resolved information is obtained according to the protocol of master–slave OCT [22]. This by calculating the product of the photodetected signals from both interferometers, and integration of values over the sweeping time, τ, the processing frequency bandwidth is largely reduced from the large frequencies present in the two signals to a maximum frequency comparable with inverse of τ. We refer to such a method as downconversion master–slave (DMS) [23]. The issue of specific processing required for forward and backward sweep is also automatically addressed as the same SS drives both interferometers. However, in order to address the dilemma of signal processing speed that is exacerbated by bidirectional sweeping, the DMS is implemented here using analogue mixing.

In order to present the DMS implementation and illustrate the nonlinearities dependent on each sweep of the SS, complex Master–Slave method [24, 25] is used, as described in the next section.

## 2 Complex Master Slave

The light source used in this work is a MEMS–VCSEL with a bidirectional sweeping rate of 1.6 MHz. The details of the source have been described in previous reports [26, 27]. In prior work, only a single sweep was used to generate OCT images, producing an effective repetition rate of 800 kHz [28]. To exploit the SS's full performance, both bidirectional sweeps are used in this study. As the tuning curves are different from forward to backward sweeps, a specific calibration must be applied for each sweep to obtain resolution limited A‐scans.

This problem is illustrated using the complex master–slave (CMS) protocol. As explained in previous reports, CMS is a phase retrieval method that allows to generate A‐scans from pre‐calculated datasets of channeled spectra [24, 25]. The CMS protocol requires a sync pulse only, employing a *λ*‐trigger at a specific wavelength and no clock. Hardware calibration uses a digitizer with clock provided by a fixed OPD interferometer. Conventional numerical procedures, such as phase calibration by dispersion compensation (PCDC), correct for nonlinearities in tuning and dispersion by resampling the data, that is, the photodetected signal corresponding to the CS. Conventional software processing is based on the phase variation over the wavenumber, employed in a suitable resampling procedure that leads to a linear in phase CS. The resampled CS is then subject to a FFT operation, that returns an A‐scan. The CMS technique does not require a linear variation of the CS phase. Using a mirror, a few experimental spectra (at least two) are acquired for different optical path differences in the interferometer. Then using these collected channeled spectra, two functions are obtained: a function *g*(*k*), which describes the nonlinear tuning, and a function *h*(*k*), related to the unbalanced dispersion in the interferometer. In the next section, we will show that due to different nonlinearities in sweeping between the two consecutive sweeps, a bidirectional sweeping laser requires more sophisticated signal processing than unidirectional sweeping lasers. The function *g*(*k*) is responsible for such differences. Once functions *g*(*k*) and *h*(*k*) are obtained, then all other shapes of channeled spectra can be inferred for any OPD using the protocol described in reference [25]. These theoretically inferred spectra are called masks they are top hat channeled spectra, which are chirped due to sweeping nonlinearities. The masks obtained are unique for each sweep hence they must be calculated for both forward and backward sweeps.

### 2.1 OCT Interferometer and Acquisition—Numerical Generation of Masks

**FIGURE 1:**
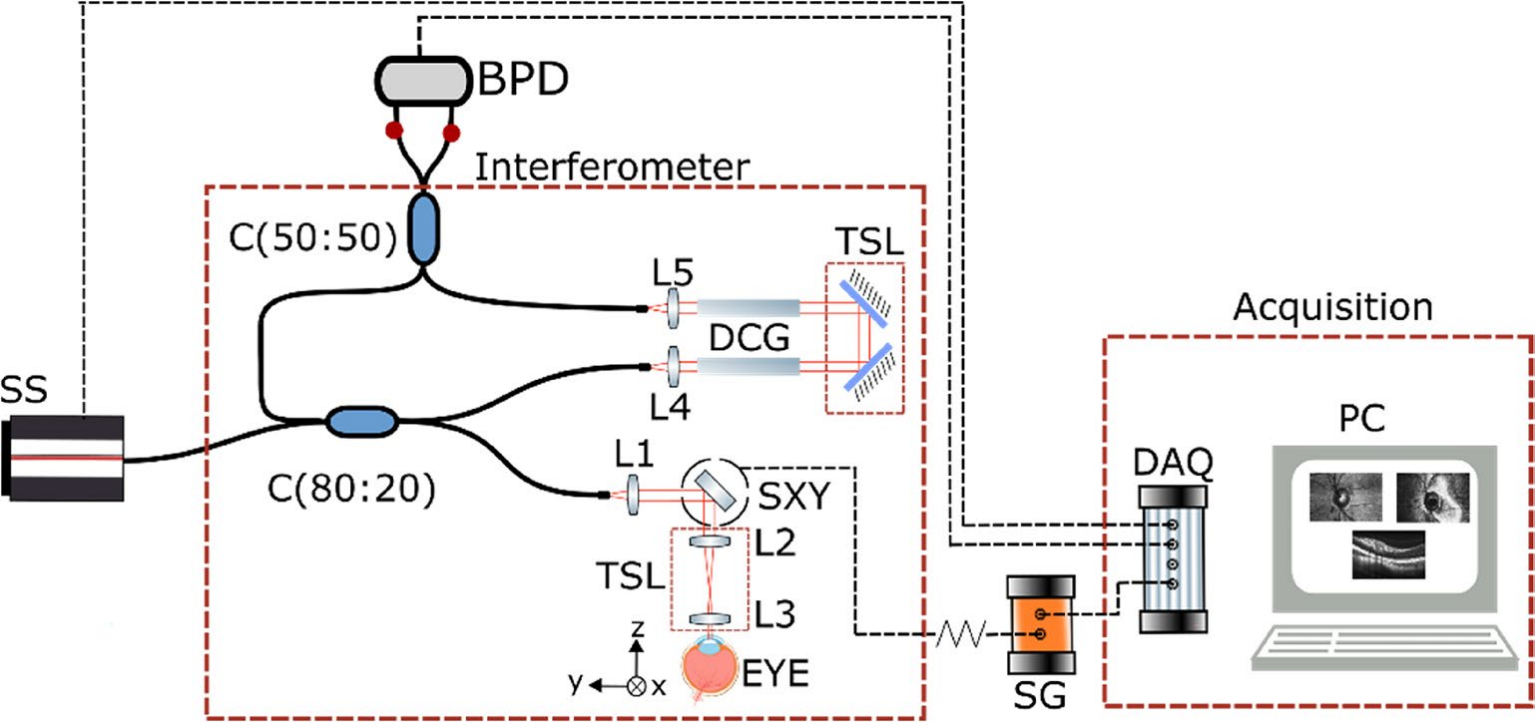
Schematic of the SS‐OCT system. SS: MEMS‐VCSEL swept source, interferometer (C: Couplers, TSL: Translation stage launcher, DCG: Dispersion compensating glass, SXY: 2‐D lateral scanning head, L1‐5: lenses), and acquisition (DAQ: digitizer, BPD: balanced photodetector, PC: computer); SG: dual signal generator.

The instrument, presented in Figure 1, is based on a Michelson interferometer composed of an 80/20 directional coupler where 20% of the light is guided toward the sample arm, and 80% toward the reference arm. The back‐scattered light from the sample interferes with light from the reference arm in a 50:50 directional coupler before reaching a balanced photodetector.

The output of the light source includes a built‐in booster that can deliver optical powers ranging from 20 to 40 mW. In the first experiment, the source was set to 20 mW. A system sensitivity of 90 dB was measured when the optical power toward the sample was 1.9 mW. In the sample arm, the light is conveyed via a galvanometer scanning head composed of 2‐D orthogonal scanners. For precise measurements, a high‐speed oscilloscope (Teledyne Lecroy, WaveMaster 820Zi‐B) was used for digitizing the signal at the output of a 23 GHz bandwidth balanced photodetector (Optilab, BPR‐23‐M). For imaging purposes, a balanced photodetector with lower noise and higher gain was employed (Thorlabs, PDB482C‐AC, 1 GHz). The photo‐detected signal was digitized at 4 GS/s using an acquisition card (AlazarTech, ATS9373). A LabVIEW software was created to enable real‐time display of the OCT images using the CMS approach. The software simultaneously displays three images: a confocal, an en‐face OCT image (C‐scan), and a cross‐section OCT image (B‐Scan). To ensure synchronization, the SS triggers the acquisition card using a TTL signal, which operates in synchronism with the signal driving the galvanometer scanners.

## 3 Results CMS

### 3.1 Digital or Numerical Master Slave: Numerical Generation of Masks

**FIGURE 2:**
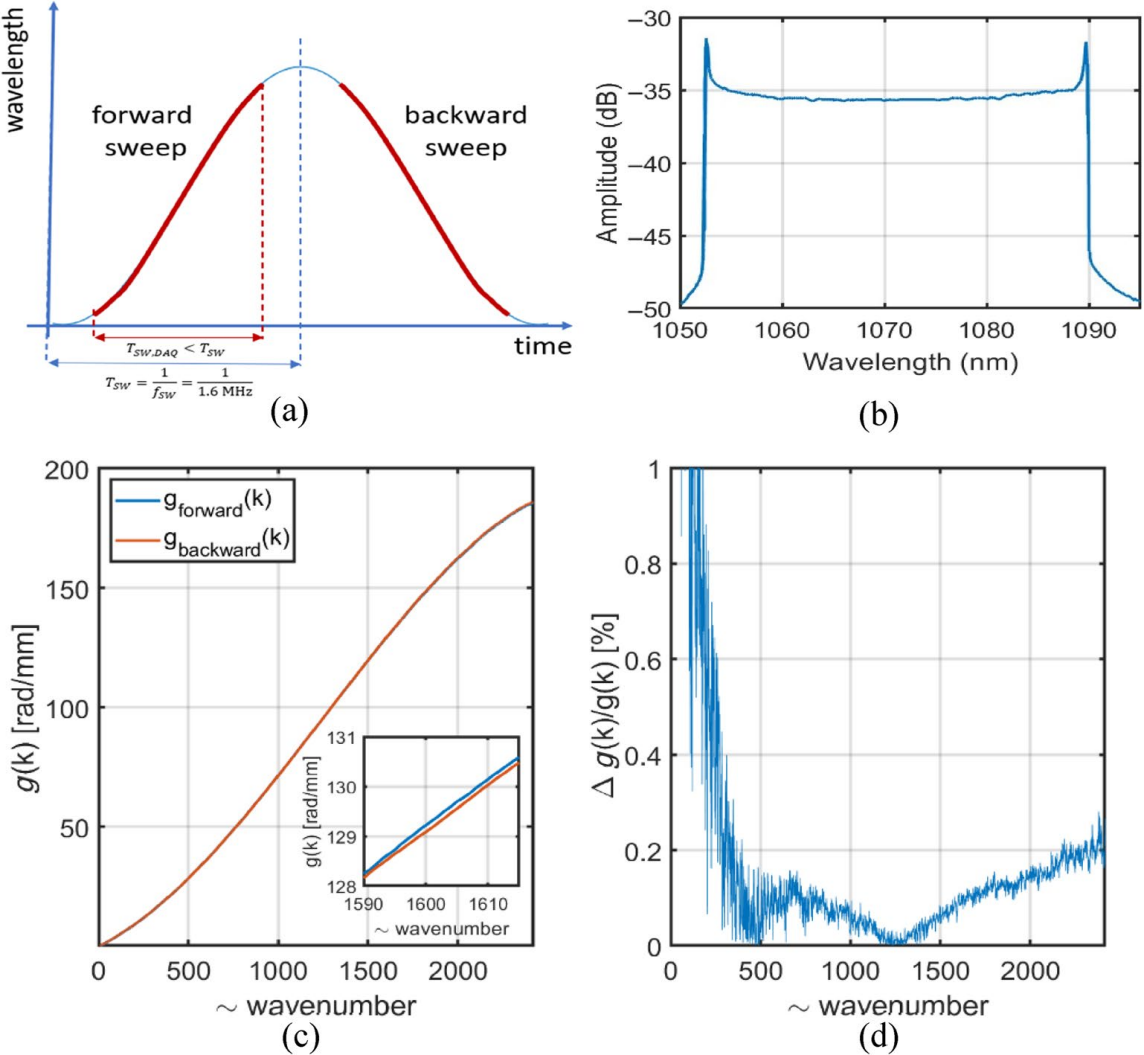
Forward and backward sketch. (a) Waveform applied to the MEMS in the swept source; (b) tuning spectrum as shown on the optical spectrum analyzer; (c) superposition of *g* function for forward and backward sweeps. (d) Differences between forward and backward sweep.

**FIGURE 3:**
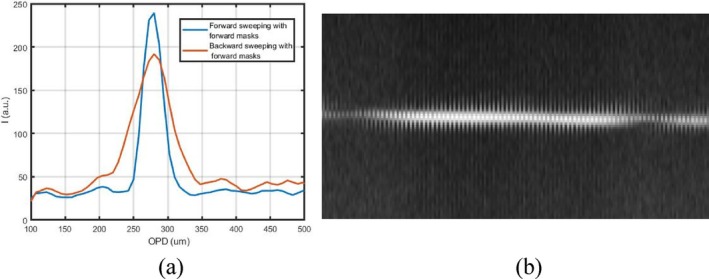
(a) A‐Scan plots of consecutive sweeps, forward and backward, where masks of the forward sweep are used for forward (blue) and backward (red) sweep; (b) B‐Scan obtained under the same conditions.

The driving signal of the MEMS–VCSEL is shown in Figure 2a. Figure 2b presents the output spectrum of the SS. Two sets of five calibration channeled spectra are acquired where each spectrum contains two channeled spectra for the two directions of tuning, forward, and backward sweeping. Then, these were split into two sets each, by suitably shifting the trigger delay from the λ‐trigger. The functions *g*(*k*) for the forward and the backward sweep are obtained from the calibration spectra. Relative differences between the *g*(*k*) functions corresponding to the two sweeps cannot be seen in Figure 2c, and are barely seen in the inset in Figure 2c. As shown in Figure 2d, the differences are minimal, for less than 1%. Although the differences are small, the A‐scans and B‐scans obtained with a mirror are corrupted if the wrong masks are used, as illustrated in Figure 3. In Figure 3a, the A‐scan profiles are affected in shape and position. Even at a relatively small OPD, the resulting axial resolution is poorer by a factor of larger than three when the incorrect set of masks are employed. The experimental axial resolution worsens from ~20 to ~70 μm on the backward sweep. This difference is even more noticeable at larger OPD values.

The coherence length of the MEMS–VCSEL used in this work can reach over 9 cm. Therefore, it is essential to process the signal to perfection to enable a comparable value for the axial range to that of the coherence length, that is, for differences between the two sweeps to not be visible even at larger OPD values. As observed in Figure 3b, the T‐scan corresponding to a mirror surface is represented by two sets of small stripes of different extensions. This demonstrates the need to perform separate signal processing on each sweep in order to make use of both sweep directions.

**FIGURE 4:**
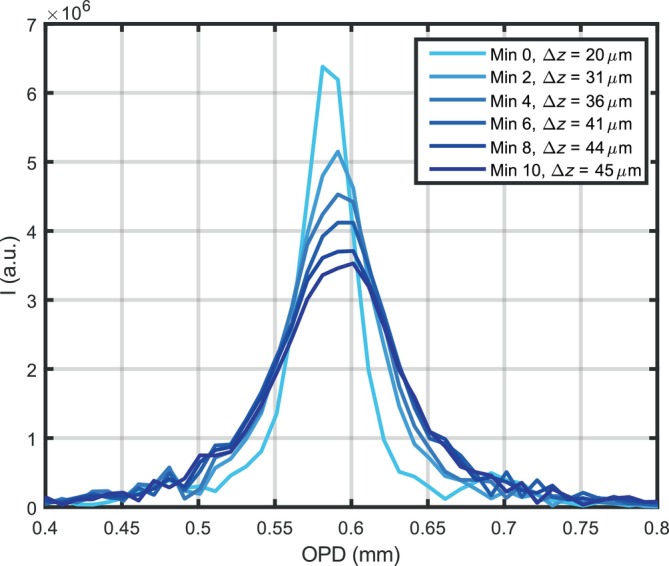
A‐Scan over time. A‐Scans produced by CMS with the same calibration files over 10 min from powering up the source.

It was observed that once *g*(*k*) and *h*(*k*) are obtained and masks calculated, the calibration obtained does not guarantee good image processing over time. In less than 10 min the thickness of the T‐Scan increases. As a consequence of these sweep‐to‐sweep variations, the output peak as a result of the initial calibration differs, as shown in Figure 4. A mirror is placed in the sample arm to obtain a sharp peak for forward sweeping with the initial calibration, and channeled spectra are obtained every 2 min until 10 min. Figure 4 shows the peak degradation over time; right after calibration the FWHM of the peak is ~20 μm, and after 10 min the FWHM evolves to ~45 μm. Moreover, the peak height drops to half, hence the sensitivity of the system falls down. In order to perform CMS based imaging is recommendable for the calibration files to be collected after a warm‐up time of 10 min. The thermal variations are then less noticeable, but some differences will still be present, unless calibration is not done.

## 4 Downconversion Master Slave

The problems signaled above in terms of different *g*(*k*) values on the two sweeps and time instabilities of *g*(*k*) are addressed in a modified master–slave configuration. The method proposed here is inspired by the master–slave protocol [22], where the mask is generated in real time using another interferometer, called as Master. In principle, many Master interferometers can be used, to produce masks corresponding to different OPD values in real time, to generate as many en‐face images as the number of Master interferometers.

In practice, to achieve DMS, the integration of the product of the two CSs is performed using a RF analogue mixer. Such mixer produces the product of the two RF signals, obtaining signals pulsating at the addition and subtraction of the frequencies of the two RF signals. The subtraction signal is called downconverted signal; hence, the method is referred to as DMS OCT. The interferometer presented in Figure 1 is now employed as the Slave interferometer and the second interferometer acts as a master interferometer, as initially described in reference [25]. Considering the object in the Slave interferometer made of scattering centers at different axial positions along the depth determining OPD values $z_{i}$, and the OPD in the master interferometer as $z_{M}$, the modulation in the two channeled spectra, Slave, 1 and Master, 2 can be written as:

(1) $$\begin{array}{c} CS_{M}=r_{M}\sin\left[g\left(k\right)\;z_{M}+h_{M}\left(k\right)\right]\;CS_{S}=\sum_{i=1}^{N}r_{i}\;\sin\left[g\left(k\right)z_{i}+h_{S}\left(k\right)\right] \end{array}$$

Then the downconversion signal, DMS, can be expressed as:

(2) $$\begin{array}{c} \mathrm{DMS}\;\left(z_{M}=z_{i}\right)=\text{filter}\left(CS_{M}\times CS_{S}\right)=r_{M}r_{i}\;\cos\left(\Delta h\left(k\right)+\phi_{\mathrm{rand}}\right) \end{array}$$

where $h_{S}\left(k\right)$ and $h_{M}\left(k\right)$ represent the uncompensated dispersion in each interferometer. The essence of downconversion lies in the fact that the factor multiplying the OPD, $z_{M}$ and $z_{i}$, is the same in both signals, *g*(*k*), as both interferometers are driven by the same SS. As a result, when both signals exhibit the same modulation periodicity, that is, they are obtained for the same imaging depth $z_{i}=z_{M}$, the output signal is at its main peak. When the two signals exhibit different periodicities, the product oscillates up and down and the integral for the duration of sweeping exhibit insignificant values. A low‐pass filter is used after the mixer to perfect the spectral integration for the sweep duration and eliminate the fast varying signals at the added frequencies.

**FIGURE 5:**
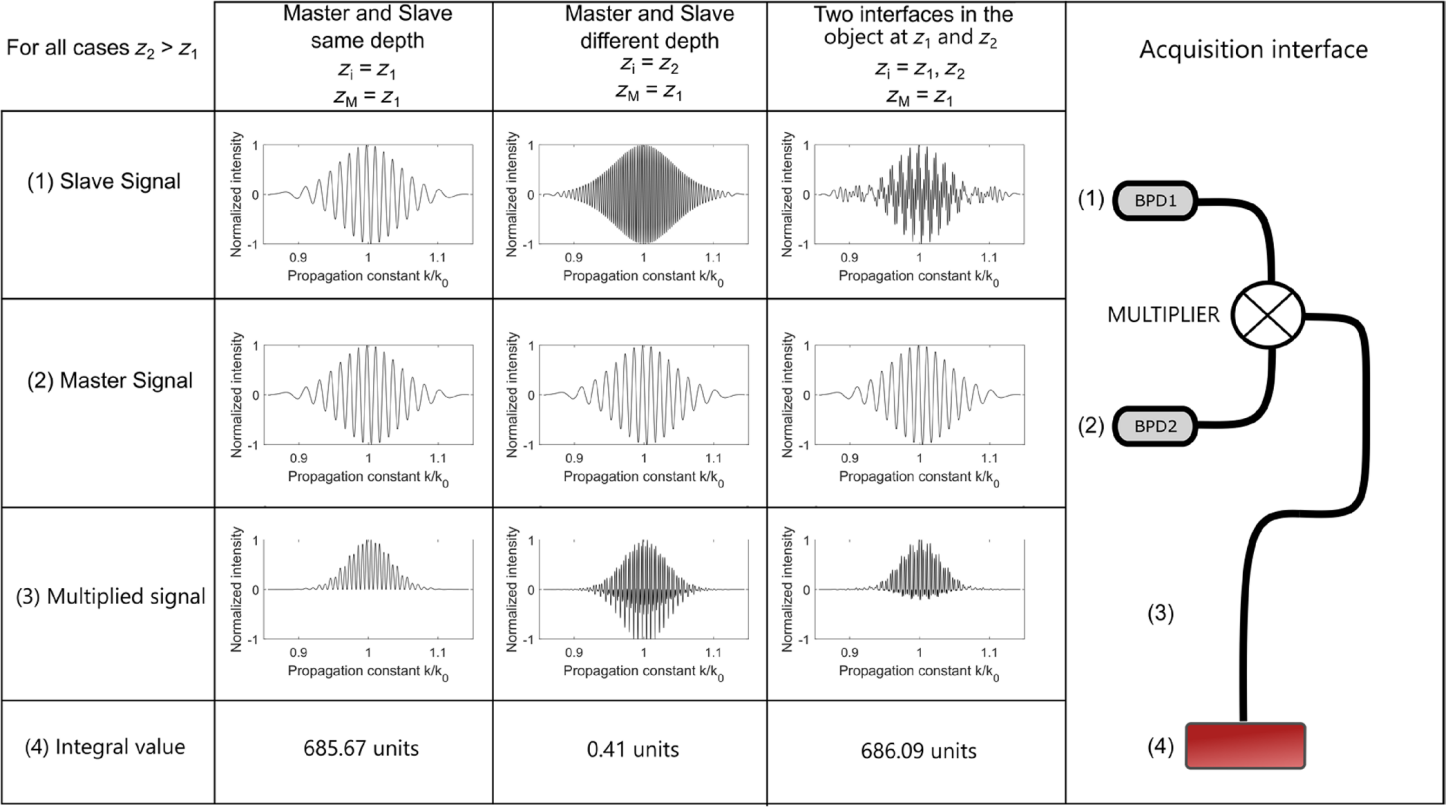
Signal evolution from the two photodetectors blocks through multiplication and integration. (1) and (2) BPD1. (2) Balanced photodetectors. (3) Multiplier. (4) Computer, signal integrator.

Figure 5 was produced from simulating the CS in Matlab using a source with central wavelength at 1060 and 40 nm bandwidth. A Gaussian shape was used for the spectrum envelope. Nonlinearities in sweeping are introduced by a cosine function in k. The same reflectivity value has been considered for two scattering centers in the object determining OPDs of $z_{1}$ and $z_{2}$. In Figure 5, the photodetected signal obtained due to the modulation of the CS at the slave interferometer output delivered by BPD1 is shown in the first row, either for a mirror placed at a distance that determines $z_{1}$ in the first column, or larger $z_{2}$ in the second column. Then the third column shows the slave signal in case of an object composed of two interfaces at $z_{1}$ and $z_{2}$. This is obtained by simple addition of channeled spectra modulations according to (1). In the second row, the signal mask delivered at the output of BPD2 by the master interferometer (2) is shown for an OPD in the master interferometer chosen to match the OPD in the slave interferometer, that is, $z_{M}=z_{1}$. Their multiplication is shown by the mixed signal (3), in the third row. The integral values of oscillations during the sweeping interval, τ, are shown at (4). The multiplied result in the third row for the same OPD in both interferometers is shown in Column 1, displaying a large DC value and the integration result calculated below as (4), of 685.67. The DMS operation recognizes in this way the existence of modulation in the CS from the slave interferometer, similar to that selected by the OPD in the master interferometer. If in the slave interferometer the interface is moved to $z_{2}$, that is, for the case in column 2, then the multiplied result exhibits oscillations up and down and the integration is much lower, 0.41. This low value means that in the CS from the slave interferometer there is no component pulsating at the modulation in the CS of the master interferometer, determined by $z_{1}$. In the last Column 3, third row, again, DMS recognizes existence of modulation for OPD selected in the Master interferometer, for an object consisting in two interfaces, at $z_{1}$ and $z_{2}$, by a large value of the integration calculation.

A low‐pass filter cleans the signal. Its cut‐off frequency should be according to Nyquist 2/τ, in order to conserve the bandwidth of lateral scanning over each pixel [29]. DMS delivers significant DC components only for mixing signals that differ in frequency by less than the bandwidth of the low pass filter, that is, favoring signal from the depth corresponding to the OPD used to infer the mask. The strength of the result of such an integration is nothing else but the amplitude of the A‐scan for the depth in the sample selected by the OPD value of the mask.

### 4.1 Interferometers and Display—Real‐Time Generation of Masks

**FIGURE 6:**
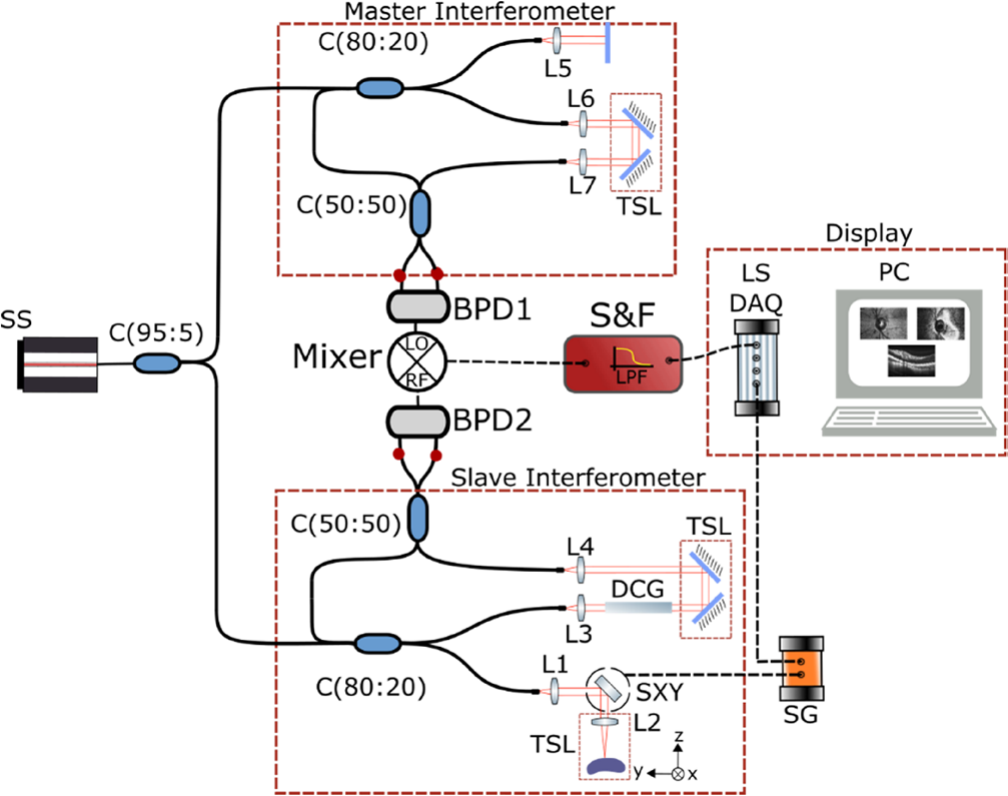
Schematic of the DMS‐OCT system. SS: MEMS‐VCSEL swept source, interferometers (C: couplers, TSL: translation stage launcher, DCG: dispersion compensating glass, SXY: 2‐D lateral scanning head, L1‐8: lenses, DCG: dispersion compensating glass), and display (LS DAQ: slow digitizer, BPD: balanced photodetector, PC: computer, S&F: signal amplifier and filter); SG: dual signal generator.

The output power from the SS is split with a broadband 95/5 coupler to the master and slave interferometer, with larger power to the slave interferometer. As seen in Figure 6, both interferometers use recirculation of the reference path, employing a broadband directional coupler, 20/80. In both interferometers, 80% is sent to the recirculating reference path. The light from the two arms is then recombined in a 50/50 coupler, whose outputs are connected to balanced photodetectors, BPD1 (Thorlabs, PDB482C‐AC, 1 GHz). The two interferometers are similar, with the only difference being that of a lateral 2D scanner, which consists of two orthogonal galvo‐scanners in the object arm of the slave interferometer. Lens L1 is a 3 cm focal length (Thorlabs, AC254‐030‐B), and L2‐L7 are aspheric objective lenses (Newport, 5724‐C‐H). Glass rods minimize the unbalanced dispersion between the two interferometers. The RF electrical signals from BPD1, the master, and from BPD2, the slave, are sent toward the local oscillator (LO) and RF inputs, respectively, of a double balanced passive mixer (Minicircuits, ZFM‐4‐S+). The output signal is amplified and filtered using a filtered preamplifier, S&F (Stanford Research, SR560). The output signal from S&F is then digitized with a slow digitizer (NI PCI 6132, 2.5 MSa/s) and the low frequency signal is displayed in form of an en‐face OCT image. A custom software delivers a real‐time en‐face image, where the number of lines in the frame, speed of the scanners, and gain in the images can be modified. In conventional Fourier transform based OCT, where a FFT processor is used, the signal from the OCT interferometer is multiplied numerically with that of multiple harmonics; here, the signal from the slave interferometer is multiplied with the signal generated by the master interferometer using a RF mixer and the role of harmonics is replaced by the chirped signal generated by the master interferometer.

## 5 Results DMS

### 5.1 Downconversion Master Slave: Real‐Time Generation of Masks

With the master interferometer set at a chosen OPD value, the axial resolution can be estimated by changing the OPD in the slave interferometer when the object is a single surface reflector (mirror). This procedure is different from Figure 1, where the axial resolution is obtained from the FFT or MS protocol giving an A‐scan peak, whose width along the axial extension of the A‐Scan is measured. Two measurements are presented.

**FIGURE 7:**
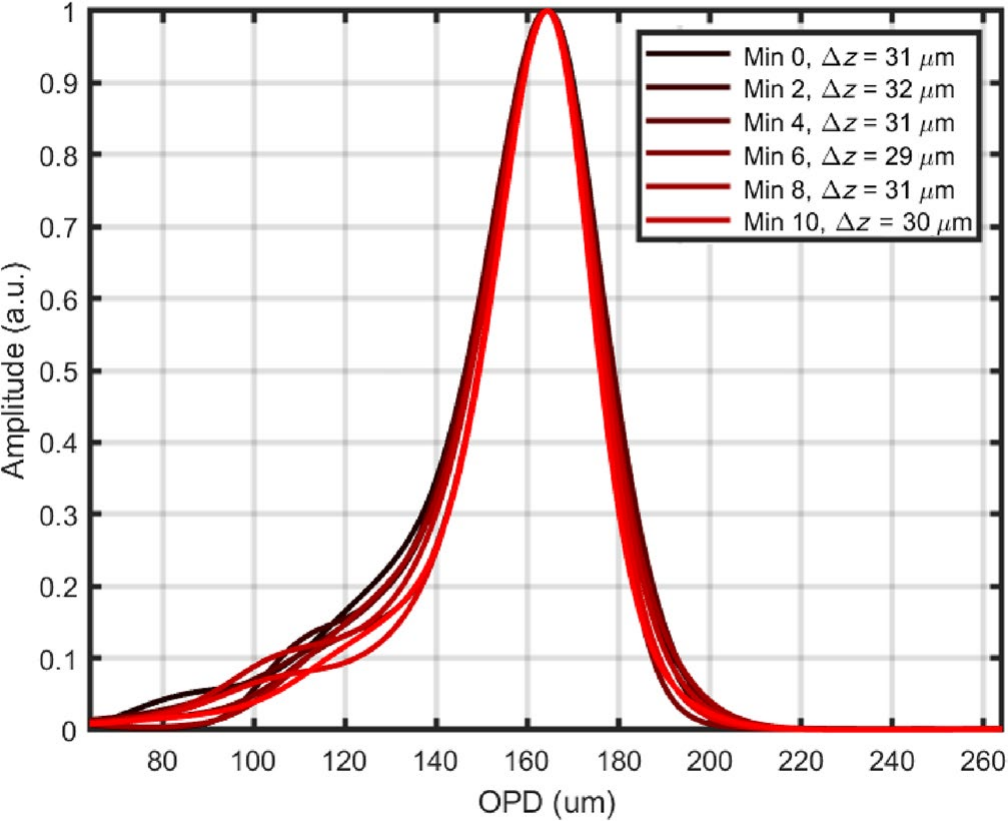
Stability of the axial resolution. Output of the beating signal measured through an RF analyser. Six graphs superposed acquired at a 2 min interval between them.

In a first method, the axial resolution was measured from the RF spectrum analyser of the mixer signal, close to |OPDM‐OPDS| = 0 where the OPD in the master interferometer is OPDM and the OPD in the slave interferometer is OPDS. For this, the lateral scanning was stopped and on a span of 30 MHz, we first identified the relation between OPD and frequency, corresponding to 12.8 μm/MHz. From this conversion, the width of the peak generated from the mixer is measured to be 5 MHz corresponding to 32 μm axial resolution along the depth coordinate, as shown in Figure 7. In order to evaluate the time behavior of the axial resolution, the RF signal was recorded in intervals of 2 min during a total span of 10 min. The RF signal is composed of multiple peaks spaced at 1.6 MHz. For proper localization of the peak, the curve has been smoothed over the maxima of multiple peaks. As illustrated in Figure 7, tolerance to inter‐sweep variations has been obtained, as shown by consistent axial resolution over time, even during the initial time interval of the laser's thermal stabilization.

**FIGURE 8:**
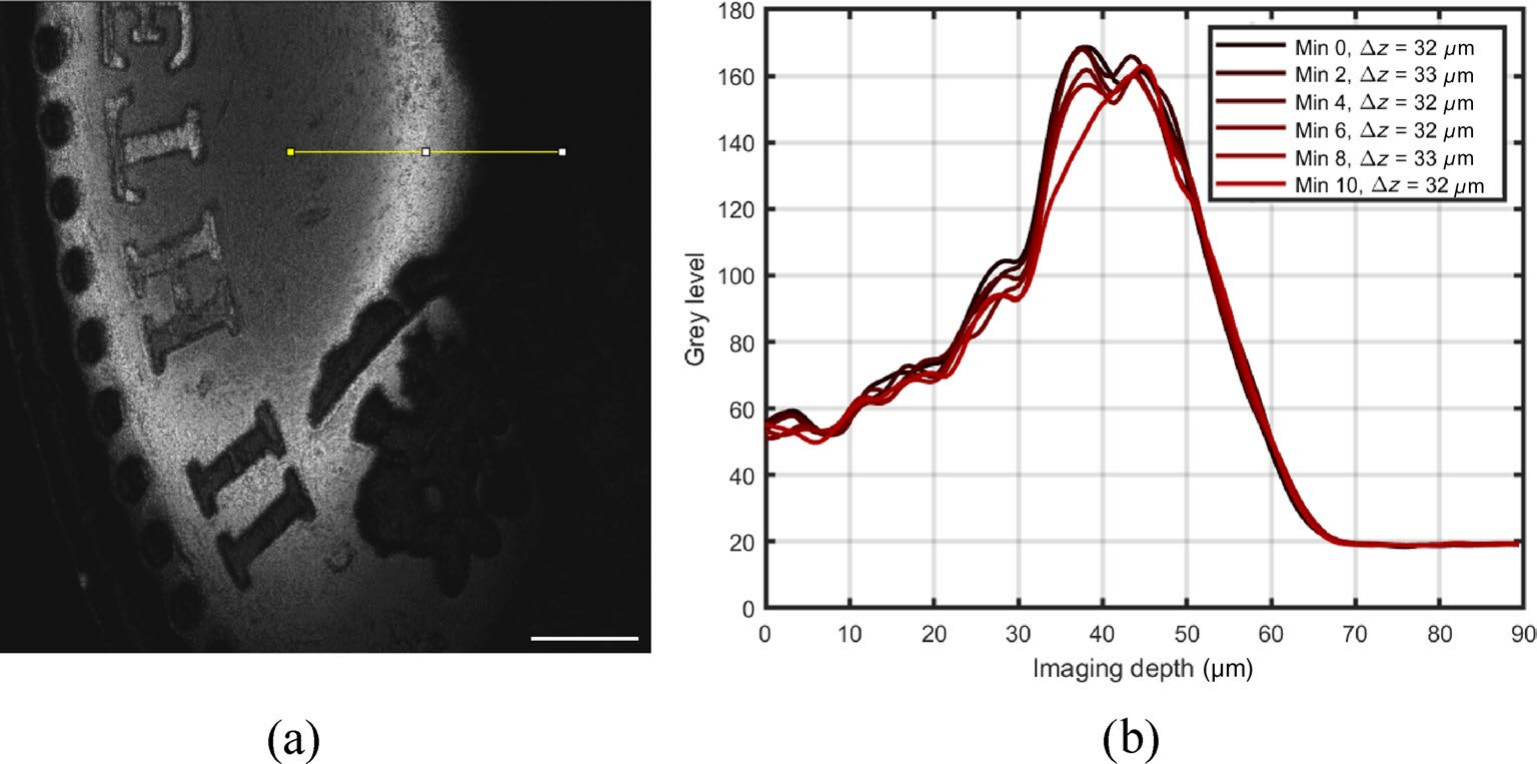
(a) En‐face image of a coin where the part selected by the coherence gate is seen. (b) Presentation of the signal strength over the yellow line. A total of 10 graphs superposed acquired at a 2 min interval between images. Scale bar 1 mm, image size 6.09 mm × 6.09 mm.

In a second method, the axial resolution is inferred from the projection of the coherence gate over the en‐face OCT image of a tilted single surface object. Figure 8a shows the en‐face OCT image of a coin. A plain section on the coin surface was used. The coherence gate width was evaluated along the yellow line displayed on the image. The brightness variation along the yellow line in the OCT en‐face in Figure 8a is represented in Figure 8b. A set of six consecutive images have been acquired every 2 min over a period of 10 min. Overall the axial resolution measured by the systems is Δz=32μm on average.

In both experiments, it can be highlighted that the effect of sweep variations on the tunning curves does not alter the resolution. The axial resolution in the DMS system drops slightly below the bandwidth limited resolution of the source. In fact, the unmatched uncompensated dispersion between the master and slave interferometer degrades the resolution, which is a disadvantage of the DMS method. Hereby, the differences between the theoretical and experimental values are strongly dependent to the mentioned mismatch. The sensitivity was measured with 1.9 mW in the sample arm, obtaining 72 dB.

**FIGURE 9:**
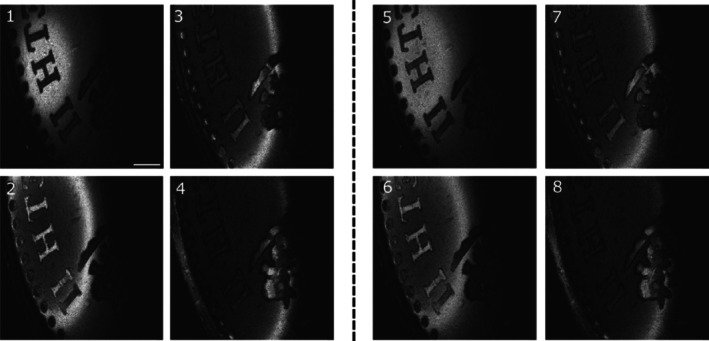
En‐face images generated by DMS. Each set of en‐face OCT images are collected at four different OPD values in the master interferometer. (1)–(4) en‐face images at *f* = 300 MHz in the master interferometer, OPD = 3.84 mm, (5)–(8) en‐face images at *f* = 600 MHz in the slave interferometer, OPD = 7.68 mm. Scale bar 1 mm, image size 6.09 mm × 6.09 mm.

The functionality of the system is demonstrated with example of images collected. To this goal, the interferometer in Figure 1 is used as slave with the master interferometer of Figure 6 images of a coin are shown in Figure 9. The fast galvanometer scanner is driven at 500 Hz and 7.8 Vpp with a triangular signal. This means that each T‐scan is obtained at 1 kHz. The slow scanner moves through 512 points in the orthogonal direction, driven by a sawtooth signal. The voltage applied is equivalent to a lateral size of 6.10 mm using a 3 cm focal length lens. Both ramps of the fast galvanometer scanner are used, together the fast and slow scanner form a square image. The following images are generated with 1.9 mW on the sample. Figure 9 shows 4 en‐face images for different values of the OPD in the master interferometer, stepped at 100 μm in panels 1–4, acquired at *f* = 300 MHz corresponding to an OPD = 3.84 mm, in both interferometers. In panels (5)–(8), the images are repeated at a frequency of 600 MHz, which corresponds to an OPD of 7.68 mm. The coherence gate is visible in both sets of images. During the imaging process, the position of the translation stage in the reference arm of the master interferometer was manually adjusted.

**FIGURE 10:**
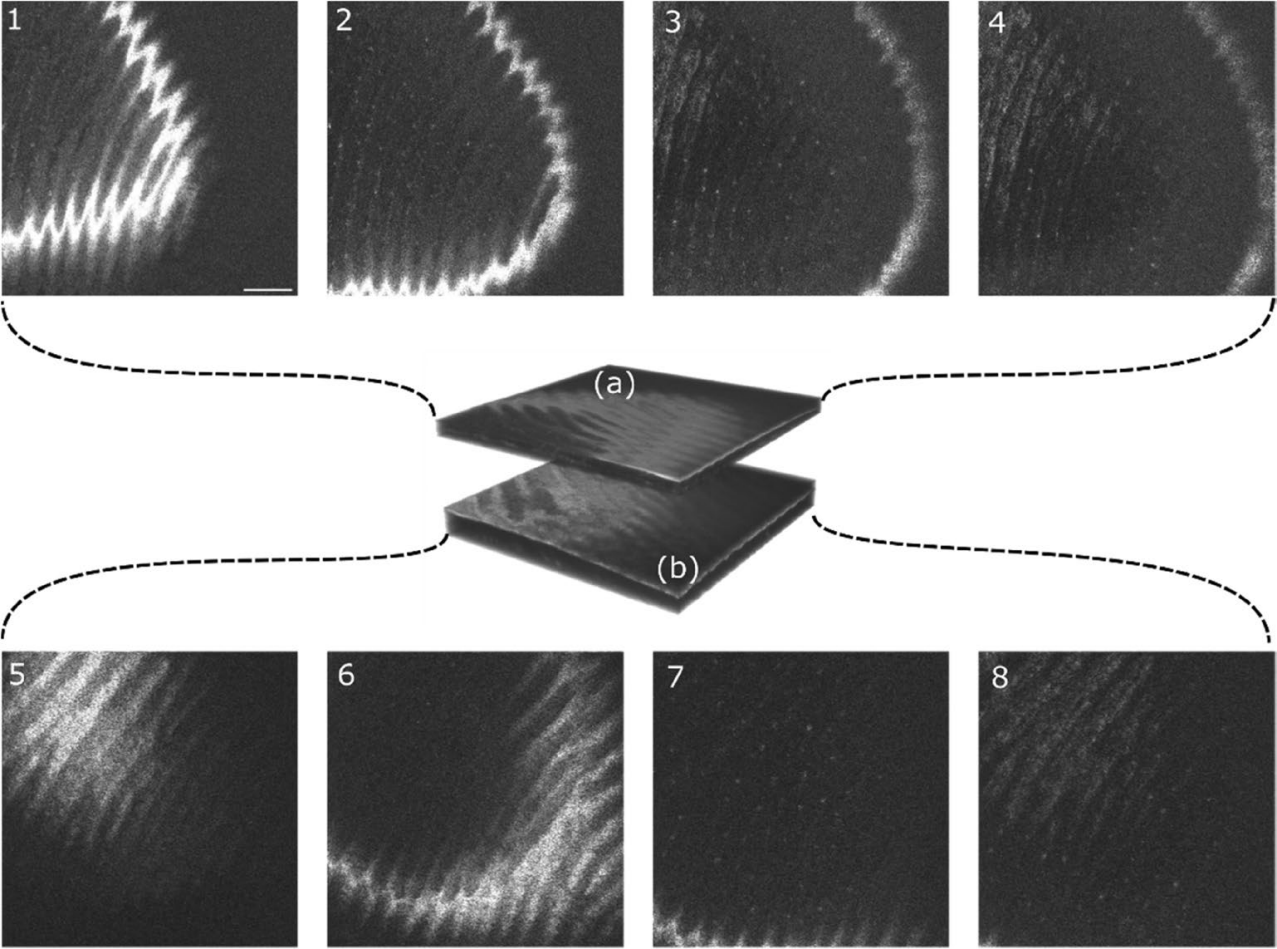
In vivo en‐face images generated by DMS. Each row represents a set of images at significant different OPD. (1)–(4) En‐face images generated at an OPD correspondent to 3.84 mm and *f* = 300 MHz. (a) Volume representation of the consecutive en‐face images acquired around 3.84 mm OPD. (5)–(8) En‐face images generated at OPD = 12.8 mm and *f* = 1000 MHz. (b) Volume representation of the consecutive en‐face images acquired around 12.8 mm OPD. Scale bar 1 mm, image size 6.09 mm × 6.09 mm.

In order to demonstrate the capabilities of our system against moving objects, we imaged a human thumb. The power of the SS was increased to the point where sample plane receives 2.9 mW. The scanning speed was increased to 1 kHz for the fast scanner while preserving the same number of lines. As a consequence, the frame rate was doubled to 10 Hz. The image size remained constant during the imaging session with 7.8 Vpp. Since both ramps of the galvo scanners are used, an effective T‐Scan is produced at 2 kHz. The first set shown in Figure 10 (1)–(4) is generated at *f* = 300 MHz, and the second set (5)–(8) at *f* = 1000 MHz. The two sets were acquired at different times. The white band seen in Figure 10 (1) corresponds to the coherence gate through the fingerprint, sampling the stratum corneum. As the OPD is varied in the master interferometer, the coherence gate moves through the finger. In Figure 10 (2) the sweat ducts are visible on the left side of the image. At a deeper layer, the epidermis can be observed on the left side of the Figure 10 (4). The volume represented in Figure 10a,b, is an illustration of volumes reconstructed after scrolling through the reference stage in the master interferometer, with both sets at different OPDs. Volume reconstruction is done in post‐processing, where the images have been registered axially. Although the source allows for larger OPD, the photodetector used here limits the system's maximum axial range. A higher frequency balanced photodetector may extend the capabilities of this system.

**FIGURE 11:**
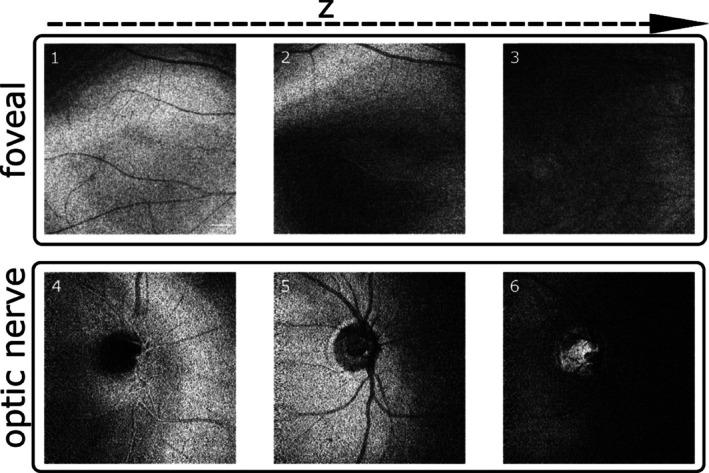
In vivo en‐face eye images generated by DMS. (1)–(3) En‐face images of the foveal area, generated at an OPD corresponding to 1.92 mm and *f* = 150 MHz (4)–(6) En‐face images of the optic nerve area, generated at an OPD correspondent to 1.92 mm and *f* = 150 MHz. Scale bar 1 mm, image size 9.3 mm × 9.3 mm.

Moreover, the slave interferometer is adapted for eye imaging. A chin rest is used to stabilize the patient head and limit movement. Power was adjusted to 1.8 mW in compliance with the ANSI standard when imaging the eye [30]. The fast galvanometer scanner runs at 1 kHz and the slow galvanometer scanner at 4 Hz, both driven by 3.4 Vpp. This represents a square image of 9.3 mm × 9.3 mm lateral size. For this set of images, the master interferometer was set to f=150 MHz, corresponding to an OPD of 1.92 *mm*. In Figure 11 (1)–(3) three different en‐face images of the foveal area are presented at different depths, showing that despite poorer depth resolution due to dispersion mismatch, sufficient axial resolution capability is demonstrated in vivo by the DMS system. The same occurs in the consecutive images in depth in Figure 11 (4)–(6) from the optic nerve. The coherence gate is moved axially by actuating on the reference arm of the slave interferometer. In this way, the visualization of deeper layers is possible. This can be seen in Figure 11 (4) and (6), while in Figure 11 (6), the lamina cribrosa is seen bright. In this figure the other pixels are closer to the anterior part of the eye, well outside the depth of the lamina, not within the axial resolution interval, hence they appear as dark.

## 6 Comparing MS Procedures

**FIGURE 12:**
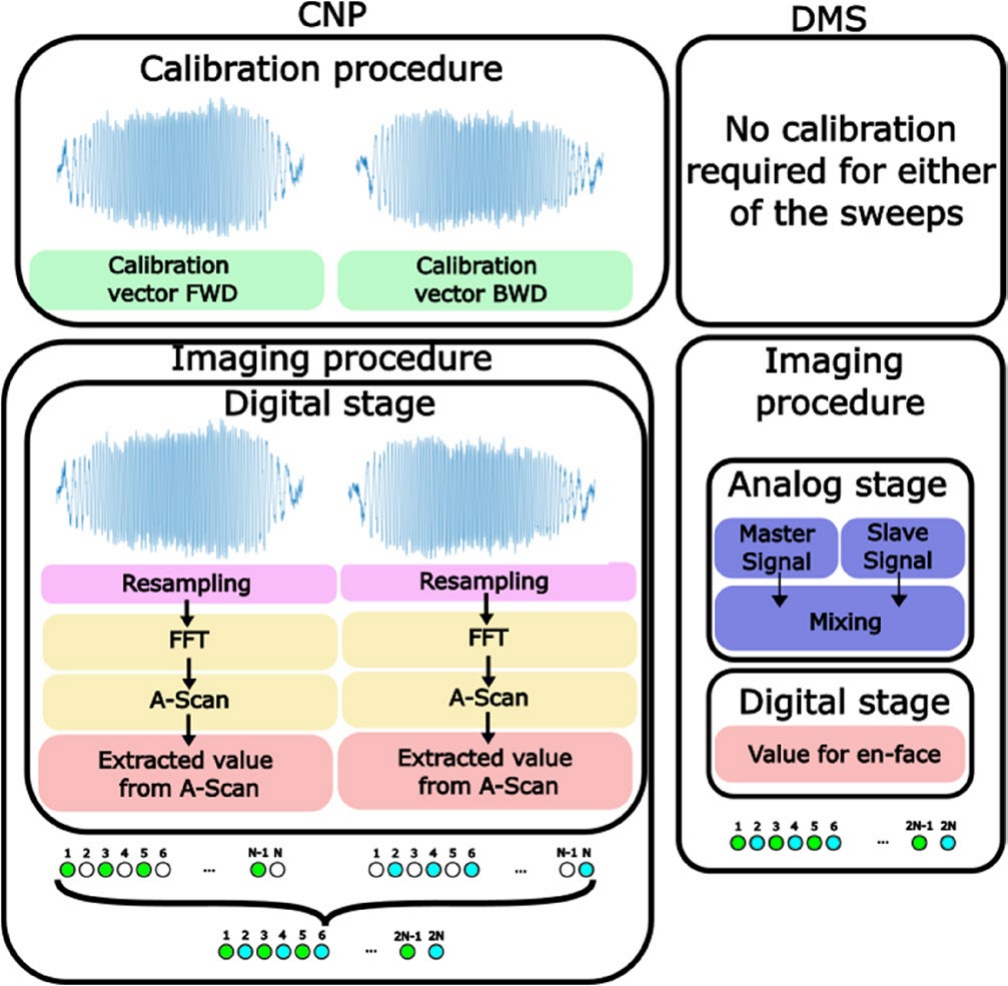
Comparison of conventional numerical protocol (left) and downconversion (right). Green and blue dots meaning sweep was used, forward and backward, respectively. White dot meaning, no sweep was used while having the sweep.

Figure 12 provides a flow chart graphical representation of the two methods under consideration, namely the conventional numerical protocol (CNP) and DMS. As such, the methods mentioned in the introduction based on subsampling are categorized as CNP. On the left, conventional numerical procedures (FFT or CMS) are shown; the two columns of steps illustrate the need for separate processing steps for each sweep. When using FFT, calibration handles a separate vector of data for backward and forward sweeping. In FFT, the calibration vector contains data resampled according to each sweep. Only after data is resampled, an FFT can be computed. For CMS, this may mean different masks. CMS uses the raw data, but requires masks to perform the CMS protocol on each sweep [25].

A major challenge when utilizing either FFT conventional method or CMS, both based on numerical calculations, is their inability to correct for changes in the sweeping parameters over time. This is because the resampling in the conventional FT based OCT as well as the calibration masks are obtained only once before the imaging step, and if the phase alters for any reason, the acquired masks will not account for these fluctuations. Thus, sweeping stabilization is typically necessary when utilizing computational phase methods, which can possible be achieved by using a clock. This possibility could not be tested here as no clock was available. However, in the case of DMS, the sweep changes take place in both interferometers at the same time, resulting in no degradation of their mixing over time due to sweep variations. This means the “masks” are generated in real‐time for each en‐face image, enhancing the system tolerance to sweep‐to‐sweep variations.

For several‐MHz swept‐sources, digitizers are available and processed data may be still produced with a maximum delay of one lateral scan or one volume, that is, quasi real time and acceptable if the time lag is not too high. For larger than a few MHz sweeping rates, digital oscilloscopes are needed, where data are sampled at much higher speed, but data obtained is stored locally [31, 32]. This precludes any chance of real time processing, as transfer of data may require few minutes. Even if the system is perfectly designed with zero dispersion and fully linear, where no operation apart from a FFT is required, such as in *k*‐clock systems, the signal still requires digitization, plus *k*‐clock circuitry of much larger frequency.

On the left of Figure 12, M pixels are along the lateral direction, *M* = *T*/(2*τ*), where *τ* is the unidirectional sweep time, with a period of sweeping 2*τ* and a period of lateral scanning 2T. Each image has M pixels. To produce an image with both sweeps, the conventional procedure on the left requires an extra step of interlacing the two images, with an image for each sweep. The raw signal is used in the DMS, as shown in Figure 12 right, where the final image contains 2 M points from both sweeps. The difference to numerical method MS is that the masks for the depth of interest is generated in real time.

The DMS proceeds on both sweeps continuously and lateral pixels are created in the T‐scan as the lateral scanner progresses, that is, in the image, there will be alternative pixels due to each sweep (white and green in Figure 12). Interlacing the two T‐scans or frames for each sweep to create an image based on alternate sweeps is unnecessary when performing DMS. The DMS produces an en‐face view with 2 M pixels straight away, that is, the image generated contains both sweeps (with no need for an extra operation of interlacing).

## 7 Discussion

Recent developments of fast SSs that in addition exhibit narrow instantaneous spectral linewidths, determining a long axial range, demand digitizers for 10's of GHz. By employing analogue mixing, the dilemma of high‐speed digitization to be used specifically on each sweeping direction is addressed. Instead of sampling photodetected signals at GHz rate, the digitizer needs to sample at the much smaller rate comparable to that of the sweeping rate.

In respect of using a digitizer to produce images corresponding to an axial range exceeding that determined by its sampling rate, there are already reports on OCT literature. A two input digitizer, with limited sampling rate was used to extend the axial range of a SS OCT system by engaging two inputs in sampling a 0° and 90° shifted replica of the photodetected signal [33]. For objects of limited extension in depth, optical subsampling was proposed that can produce correct cross section images of the object even if the object is placed at distances several times larger than its axial extension [34]. Using frequency combs, circular ranging was reported to image single layer extended objects [35].

It is therefore interesting to place DMS reported here in the landscape of other OCT methods addressing the high sampling rates needed. In comparison with other methods that reduce the digitization burden, such as optical subsampling, DMS can generate en‐face image correctly with no superposition from any other depth of an extended object. The object can be as extended in depth as the axial range determined by the instantaneous spectral line width [23, 36, 37]. In opposition, the optical subsampling will lead to an en‐face slice with backscattered light from multiple depths outside that of interest. An en‐face OCT image from any depth can be obtained with MS by simply changing the mask file (pre‐stored from an experimentally collected CS in [22] or from a pre‐calculated mask [38] or with DMS [23] by simply changing the optical path difference in the master interferometer). Any depth means from the whole axial range determined by the spectral line width of the SS [36, 37] and not restricted to a stripe in depth as in optical subsampling.

We anticipate that DMS may become more useful for sweeping rates exceeding 10 MHz, where only digital scopes can be used to sample the multi‐GHz signal, in which case the data acquired is stored and processed offline later [32, 39]. In such cases the DMS may find an useful niche in providing real time information on the imaged sample. This may be useful in adjusting the sample in front of the interface optics and can speed up the whole process that currently requires iterative adjustments of sample position.

Overall, DMS is able to produce en‐face images without heavy computation and with low‐bandwidth electronics. In practice there are also losses, but not due to the principle, more to do with the device employed for producing the multiplication effect. As such, the passive Minicircuits, ZFM‐4‐S+ mixer has a 7 dB conversion loss. There is also cross talk between the inputs, RF and LO signals on one side and the output on the other side. Perfecting the MS in a single interferometer, that is, CMS using pre‐calculated masks and performing calculation with a digitizer leads to a sensitivity of 90 dB. Using the downconversion 72 dB was obtained. Most of the difference in sensitivity is attributed to uncompensated dispersion mismatch between interferometers. For future system designs, these issues can be amended by careful dispersion compensation, and use of electronics with lower figure noise.

There is however a degrading factor, if deterministic oscillations pulsate similarly in the two interferometers, then these determine an extra source of noise. When perfecting FFT, the harmonics are clean. When perfecting CMS (that employs pre‐calculated masks), the masks are also clean. Pulsations seen in the two interferometers in a single arm concur in creating noise by the very process of multiplication.

## 8 Conclusions

In this study, we address key challenges associated with bidirectional sweeping in OCT. Two alternative imaging approaches are compared, both based on the MS protocol. We refer to the first approach as complex master slave, where complex masks are obtained from several experimentally collected spectra followed by a numerical synthesis. Its calibration procedure replaces the step of resampling and linearization of data, widely used by the OCT community [32] before applying a Fourier transform. CMS uses masks obtained by employing the same interferometer for both calibration that is, generation of experimental spectra followed by calculation of masks as well as for imaging. Once masks are generated, A‐scans, B‐scans, and C‐scans can be obtained, similar to the FFT‐based method. (FFT based method also uses a single interferometer, for calibration with a mirror and imaging when the mirror is replaced by the object to be imaged). When driven by bidirectional sweeping lasers, we demonstrate here that even minor variations in sweep between forward and backward scans can substantially influence image quality, hence, the need for separate processing of signal obtained from each sweep direction. In addition, CMS or FFT‐based methods often require calibration if sweeping parameters vary over time. Such an instability problem is addressed using the DMS‐OCT procedure. In addition, the mask generated by DMS contains both sweeps straight away, eliminating the need for the interlacing step required by both FFT based and CMS based methods. Tolerance of instabilities in *g* comes from the generation of such masks in real‐time. As another advantage, the signal provided by DMS is of much lower bandwidth (i.e., downconverted in frequency), enabling the generation of en‐face OCT images for much higher sweeping speeds.

DMS presents a promising solution for bidirectional sweeping lasers thanks to its simplicity. This approach eliminates the need for resampling, additional calibration, or processing of the two sweeps separately. Moreover, it offers a cost‐effective implementation by simplifying the digitization process. Despite its limitations, such as generating a single en‐face OCT image and potential dispersion mismatches between the slave and master interferometers impacting axial resolution, more master interferometers can be utilized with careful interferometric design. Nevertheless, the DMS capability can potentially benefit systems such as; few‐MHz sweep rates, where computational demand is high, long axial range systems needed to capture GHz signals, systems using bidirectional sweeping, and finally conventional systems with a need for rapid en‐face display as it can be installed as an add‐on with minimal modifications to an already functional setup.

As long as calculations of FFT, resampling, phase compensation can be done within the sweeping interval, then digital processing can keep pace with sweeping and volume processing can be performed sufficiently fast to enable an en‐face cut with minimum delay (as long as the extra time of en‐face calculation is less than the duration of a volume frame, then there is no need for DMS). This is possible with ultra‐fast digitizers up to a few MHz sweeping rate as demonstrated by several reports. Here DMS may only be used as a low cost alternative, eliminating the high cost of the digitizer with an accepted compromise of limited information delivered in a single or a few en‐face OCT images, depending on how many master interferometers are assembled.

## Conflicts of Interest

AP and AB are co‐inventors of patents in the name of the University of Kent. TA and KY are involved on the company board of directors for OCTLight Aps.

## Data Availability

The data that support the findings of this study are available from the corresponding author upon reasonable request.

## References

[1] B. Potsaid , I. Gorczynska , V. J. Srinivasan , et al., “Ultrahigh Speed Spectral/Fourier Domain OCT Ophthalmic Imaging at 70,000 to 312,500 Axial Scans per Second,” Optics Express 16 (2008): 15149. 18795054 10.1364/oe.16.015149 PMC2743204

[2] O. P. Kocaoglu , T. L. Turner , Z. Liu , and D. T. Miller , “Adaptive Optics Optical Coherence Tomography at 1 MHz,” Biomedical Optics Express 5 (2014): 4186. 25574431 10.1364/BOE.5.004186 PMC4285598

[3] S. R. Chinn , E. A. Swanson , and J. G. Fujimoto , “Optical Coherence Tomography Using a Frequency‐Tunable Optical,” Source 22 (1997). 10.1364/ol.22.000340 18183195

[4] S. Grelet , A. M. Jimenez , R. D. Engelsholm , P. B. Montague , and A. Podoleanu , “40 MHz Swept‐Source Optical Coherence Tomography at 1060 nm Using a Time‐Stretch and Supercontinuum Spectral Broadening Dynamics,” IEEE Photonics Journal 14 (2022): 1.

[5] T. Mitsui , “Dynamic Range of Optical Reflectometry With Spectral Interferometry,” Japanese Journal of Applied Physics, Part 1: Regular Papers and Short Notes and Review Papers 38 (1999): 6133.

[6] R. Leitgeb , C. Hitzenberger , and A. Fercher , “Performance of Fourier Domain vs. Time Domain Optical Coherence Tomography,” Optics Express 11 (2003): 889. 19461802 10.1364/oe.11.000889

[7] J. F. De Boer , B. Cense , B. H. Park , M. C. Pierce , G. J. Tearney , and B. E. Bouma , “Improved Signal‐to‐Noise Ratio in Spectral‐Domain Compared With Time‐Domain Optical Coherence Tomography,” Optics Letters 28 (2003): 2067–2070. 14587817 10.1364/ol.28.002067

[8] M. Choma , M. Sarunic , C. Yang , and J. Izatt , “Sensitivity Advantage of Swept Source and Fourier Domain Optical Coherence Tomography,” Optics Express 11 (2003). 10.1364/oe.11.002183 19466106

[9] T. Klein , W. Wieser , L. Reznicek , A. Neubauer , A. Kampik , and R. Huber , “Multi‐MHz retinal OCT,” Biomedical Optics Express 4 (2013): 1890. 24156052 10.1364/BOE.4.001890 PMC3799654

[10] W. Draxinger , D. Theisen‐Kunde , L. Schützeck , et al., “High Speed 4D in‐Vivo OCT Imaging of the Human Brain: Creating High Density Datasets for Machine Learning Toward Identification of Malign Tissue in Real Time,” in SPIE‐The International Society for Optical Engineering (2023), 41.

[11] K. S. Park , E. Park , H. Lee , H. J. Lee , S. W. Lee , and T. J. Eom , “Phase Stable Swept‐Source Optical Coherence Tomography With Active Mode‐Locking Laser for Contrast Enhancements of Retinal Angiography,” Scientific Reports 11 (2021). 10.1038/s41598-021-95982-9 PMC8371173 34404853

[12] S. Tozburun , C. Blatter , M. Siddiqui , E. F. J. Meijer , and B. J. Vakoc , “Phase‐Stable Doppler OCT at 19 MHz Using a Stretched‐Pulse Mode‐Locked Laser,” Biomedical Optics Express 9 (2018): 952. 29541496 10.1364/BOE.9.000952 PMC5846541

[13] Y. Sasaki , M. Fujimoto , S. Yagi , S. Yamagishi , S. Toyoda , and J. Kobayashi , “Ultrahigh‐Phase‐Stable Swept Source Based on KTN Electro‐Optic Deflector Towards Doppler OCT and Polarization‐Sensitive OCT,” in Optical Coherence Tomography and Coherence Domain Optical Methods in Biomedicine XVIII, vol. 8934 (SPIE, 2014), 89342Y.

[14] J. Zhang , MEMS‐VCSEL Swept‐Source Optical Coherence Tomography for Multi‐MHz Endoscopic Structural and Angiographic Imaging, vol. 12 (The University of Texas of Austin, MIT, 2021), 2384. 10.1364/BOE.420394 PMC8086463 33996236

[15] D. D. John , C. B. Burgner , B. Potsaid , et al., “Wideband Electrically Pumped 1050‐nm MEMS‐Tunable VCSEL for Ophthalmic Imaging,” Journal of Lightwave Technology 33 (2015): 3461–3468. 26594089 10.1109/JLT.2015.2397860 PMC4651169

[16] W. Wieser , T. Klein , D. C. Adler , et al., “Extended Coherence Length Megahertz FDML and Its Application for Anterior Segment Imaging,” Biomedical Optics Express 3 (2012): 2647–2657. 23082303 10.1364/BOE.3.002647 PMC3470011

[17] J. Zhang , T. Nguyen , B. Potsaid , et al., “Multi‐MHz MEMS‐VCSEL Swept‐Source Optical Coherence Tomography for Endoscopic Structural and Angiographic Imaging With Miniaturized Brushless Motor Probes,” Biomedical Optics Express 12 (2021): 2384. 33996236 10.1364/BOE.420394 PMC8086463

[18] M. Klufts , A. M. Jiménez , S. Lotz , et al., “828 kHz Retinal Imaging With an 840 nm Fourier Domain Mode Locked Laser,” Biomedical Optics Express 14 (2023): 6493. 38420314 10.1364/BOE.504302 PMC10898573

[19] N. Huang , T. Hormel , G. Liang , et al., “Optimizing Numerical k‐Sampling for Swept‐Source Optical Coherence Tomography Angiography,” Optics Letters 49 (2024): 1201. 38426973 10.1364/OL.518720

[20] X. Attendu and R. M. Ruis , “Simple and Robust Calibration Procedure for k‐Linearization and Dispersion Compensation in Optical Coherence Tomography,” Journal of Biomedical Optics 24 (2019): 1. 10.1117/1.JBO.24.5.056001 PMC6992960 31087833

[21] S. Chen , B. Potsaid , Y. Li , et al., “High Speed, Long Range, Deep Penetration Swept Source OCT for Structural and Angiographic Imaging of the Anterior eye,” Scientific Reports 12 (2022). 10.1038/s41598-022-04784-0 PMC8770693 35046423

[22] A. G. Podoleanu and A. Bradu , “Master–Slave Interferometry for Parallel Spectral Domain Interferometry Sensing and Versatile 3D Optical Coherence Tomography,” Optics Express 21 (2013): 19324. 23938849 10.1364/OE.21.019324

[23] A. Podoleanu , R. Cernat , and A. Bradu , “Down‐Conversion en‐Face Optical Coherence Tomography,” Biomedical Optics Express 10 (2019): 772. 30800514 10.1364/BOE.10.000772 PMC6377877

[24] A. Bradu , N. M. Israelsen , M. Maria , et al., “Recovering Distance Information in Spectral Domain Interferometry,” Scientific Reports 8 (2018): 15445. 30337645 10.1038/s41598-018-33821-0 PMC6194011

[25] S. Rivet , M. Maria , A. Bradu , T. Feuchter , L. Leick , and A. Podoleanu , “Complex Master Slave Interferometry,” Optics Express 24 (2016): 2885. 26906857 10.1364/OE.24.002885

[26] T. Ansbaek , I. S. Chung , E. S. Semenova , and K. Yvind , “1060‐nm Tunable Monolithic High Index Contrast Subwavelength Grating VCSEL,” IEEE Photonics Technology Letters 25 (2013): 365–367.

[27] T. Ansbaek , I. S. Chung , E. S. Semenova , O. Hansen , and K. Yvind , “Resonant MEMS tunable VCSEL,” IEEE Journal on Selected Topics in Quantum Electronics 19 (2013): 1702306.

[28] E. A. Proano Grijalva , A. Martínez Jiménez , A. Bradu , et al., “Novel 1.6 MHz Swept Source for Real‐Time Volumetric in‐Vivo OCT Imaging of the Human Retina,” in Optical Coherence Tomography and Coherence Domain Optical Methods in Biomedicine XXVII, eds. J. A. Izatt and J. G. Fujimoto (SPIE, 2023), 8.

[29] A. G. Podoleanu , M. Seeger , G. M. Dobre , D. J. Webb , D. A. Jackson , and F. W. Fitzke , “Transversal and Longitudinal Images From the Retina of the Living Eye Using low Coherence Reflectometry,” Journal of Biomedical Optics 3 (1998): 12. 23015001 10.1117/1.429859

[30] ANSI , American National Standard for Safe Use of Lasers (Laser Institute of America, 2007).

[31] D. Huang , F. Li , Z. He , Z. Cheng , C. Shang , and P. K. A. Wai , “400 MHz Ultrafast Optical Coherence Tomography,” Optics Letters 45 (2020): 6675. 33325868 10.1364/OL.409607

[32] A. M. Jimenez , S. Grelet , V. Tsatourian , P. B. Montague , A. Bradu , and A. Podoleanu , “400 Hz Volume Rate Swept‐Source Optical Coherence Tomography at 1060 nm Using a KTN Deflector,” IEEE Photonics Technology Letters 34 (2022): 1277–1280.

[33] Y. Shi , J. Liu , Z. Gong , C. Burger , V. Jayaraman , and R. K. Wang , “Multi‐Channel Delay Sampling to Extend Imaging Depth in High‐Speed Swept‐Source OCT Systems,” Optics Letters 49 (2024): 2217. 38691683 10.1364/OL.517493 PMC11275917

[34] M. Siddiqui and B. J. Vakoc , “Optical‐Domain Subsampling for Data Efficient Depth Ranging in Fourier‐Domain Optical Coherence Tomography,” Optics Express 20 (2012): 17938. 23038343 10.1364/OE.20.017938 PMC3601603

[35] M. Siddiqui , A. S. Nam , S. Tozburun , N. Lippok , C. Blatter , and B. J. Vakoc , “High‐Speed Optical Coherence Tomography by Circular Interferometric Ranging,” Nature Photonics 12 (2018): 111–116. 29657576 10.1038/s41566-017-0088-x PMC5894866

[36] M. J. Marques , S. Rivet , A. Bradu , and A. Podoleanu , “Complex Master‐Slave for Long Axial Range Swept‐Source Optical Coherence Tomography,” OSA Continuum 1 (2018): 1251.

[37] M. J. Marques , R. Cernat , J. Ensher , A. Bradu , and A. Podoleanu , “Akinetic Swept‐Source Master–Slave‐Enhanced Optical Coherence Tomography,” Photonics 8 (2021).

[38] S. Caujolle , R. Cernat , G. Silvestri , et al., “Speckle Variance OCT for Depth Resolved Assessment of the Viability of Bovine Embryos,” Biomedical Optics Express 8 (2017): 5139. 29188109 10.1364/BOE.8.005139 PMC5695959

[39] X. Wei , A. K. S. Lau , Y. Xu , K. K. Tsia , and K. K. Y. Wong , “28 MHz Swept Source at 10 μm for Ultrafast Quantitative Phase Imaging,” Biomedical Optics Express 6 (2015): 3855. 26504636 10.1364/BOE.6.003855 PMC4605045

